# Tongxinluo Protects against Pressure Overload–Induced Heart Failure in Mice Involving VEGF/Akt/eNOS Pathway Activation

**DOI:** 10.1371/journal.pone.0098047

**Published:** 2014-06-02

**Authors:** Bo Wang, Qing Yang, Wen-wu Bai, Yi-fan Xing, Xiao-ting Lu, Yuan-yuan Sun, Yu-xia Zhao

**Affiliations:** 1 Department of Traditional Chinese Medicine, Qilu Hospital of Shandong University, Jinan, China; 2 Institute of Pathogen Biology, Shandong University, Jinan, China; 3 Key Laboratory of Cardiovascular Remodeling and Function Research, Qilu Hospital of Shandong University, Jinan, China; Macau University of Science and Technology, Macau

## Abstract

**Background:**

It has been demonstrated that Tongxinluo (TXL), a traditional Chinese medicine compound, improves ischemic heart disease in animal models via vascular endothelial growth factor (VEGF) and endothelial nitric oxide synthase (eNOS). The present study aimed to investigate whether TXL protects against pressure overload–induced heart failure in mice and explore the possible mechanism of action.

**Methods and Results:**

Transverse aortic constriction (TAC) surgery was performed in mice to induce heart failure. Cardiac function was evaluated by echocardiography. Myocardial pathology was detected using hematoxylin and eosin or Masson trichrome staining. We investigated cardiomyocyte ultrastructure using transmission electron microscopy. Angiogenesis and oxidative stress levels were determined using CD31 and 8-hydroxydeoxyguanosine immunostaining and malondialdehyde assay, respectively. Fetal gene expression was measured using real-time PCR. Protein expression of VEGF, phosphorylated (p)-VEGF receptor 2 (VEGFR2), p–phosphatidylinositol 3-kinase (PI3K), p-Akt, p-eNOS, heme oxygenase-1 (HO-1), and NADPH oxidase 4 (Nox4) were measured with western blotting. Twelve-week low- and high-dose TXL treatment following TAC improved cardiac systolic and diastolic function and ameliorated left ventricular hypertrophy, fibrosis, and myocardial ultrastructure derangement. Importantly, TXL increased myocardial capillary density significantly and attenuated oxidative stress injury in failing hearts. Moreover, TXL upregulated cardiac nitrite content and the protein expression of VEGF, p-VEGFR2, p-PI3K, p-Akt, p-eNOS, and HO-1, but decreased Nox4 expression in mouse heart following TAC.

**Conclusion:**

Our findings indicate that TXL protects against pressure overload–induced heart failure in mice. Activation of the VEGF/Akt/eNOS signaling pathway might be involved in TXL improvement of the failing heart.

## Introduction

Heart failure is a major cause of mortality worldwide. A common cause of heart failure is chronic pressure overload due to hypertension or aortic stenosis, which leads to cardiac hypertrophy that may progress to heart failure [1]. Heart failure has a complex phenotype that includes reduced cardiac diastolic and contractile function, increased myocyte cell death, and myocardial fibrosis. Although the mechanism mediating the pathological changes responsible for heart failure has not been fully elucidated, experimental evidence suggests that disruption of coordinated tissue growth and angiogenesis may contribute to the development of pressure overload–induced cardiac hypertrophy and heart failure [2], [3]. In mice, myocardial capillary density is decreased at the early stage of transverse aortic constriction (TAC) [4]. It has been reported that insufficient angiogenic response to myocardial hypoxia leads to cardiac dysfunction [5]. Vascular endothelial growth factor (VEGF) is the main regulator of angiogenesis. In rats, gene therapy of *VEGF* overexpression ameliorates angiotensin II–induced diastolic dysfunction by promoting angiogenesis and anti-inflammation function [3]. However, the clinical safety of gene therapy has not been determined.

Tongxinluo (TXL) is a traditional Chinese medicine compound that has been used to treat angina pectoris in the clinic for the past 18 years. Using rabbits, we previously demonstrated that TXL enhances the stability of vulnerable plaques dose-dependently through lipid-lowering, anti-inflammatory, and antioxidant effects [6]. In pigs, TXL reduces myocardial no-reflow and ischemia/reperfusion injury by stimulating endothelial nitric oxide (NO) synthase (eNOS) phosphorylation via the protein kinase A pathway [7]. It has been demonstrated that NO is effective against hypertrophy and for inhibiting cardiac remodeling [8]. It has been reported that augmented eNOS signaling by drugs such as angiotensin-converting enzyme inhibitors [9], statins [10], and beta-blockers [11] is associated with improvement of heart failure. Previously, we demonstrated that TXL ameliorates cardiac remodeling after myocardial infarction in mice by promoting angiogenesis by enhancing VEGF levels and eNOS phosphorylation [12]. However, it is not known whether TXL has an effect on heart failure. We hypothesized that TXL may protect against heart failure involving VEGF/eNOS pathway activation. To test this idea, we evaluated the effects of long-term treatment with different doses of TXL on cardiac remodeling and function in a murine model of ventricular pressure overload induced by TAC surgery, and attempted to determine the possible mechanisms of action.

## Materials and Methods

### Preparation of TXL Ultrafine Powder

TXL ultrafine powder was obtained from Yiling Pharmaceutical (Shijiazhuang, China). The herbal drug was authenticated and standardized to marker compounds according to the Chinese Pharmacopoeia 2005 [13]. The components of the TXL powder and detailed preparation methods have been described previously [6].

### Ethics Statement

All animal studies were approved by the Ethics Committee of Shandong University (No. 011 in 2011 for Animal Ethics Approval) and all efforts were made to minimize suffering.

### Animals and Experimental Protocols

We used wild-type male C57BL/6 mice (9 weeks old; Vital River Laboratory, Beijing, China). The mice were randomly assigned to four groups prior to TAC surgery (n = 15 per group): Sham, TAC, TAC with low-dose TXL (TAC+TL), and TAC with high-dose TXL (TAC+TH). Mice in the latter three groups underwent TAC surgery. On day 3 post-surgery, the TAC+TL and TAC+TH groups were fed orally with 0.38 g·kg^−1^·d^−1^ and 1.5 g·kg^−1^·d^−1^ TXL ultrafine powder, respectively, for 12 weeks. The Sham and TAC groups were fed orally with equal volumes of physiological saline once daily for 12 weeks.

### TAC

Mice were subjected to TAC-induced pressure overload as previously described [14], [15]. Briefly, the mice were anesthetized with ketamine (20 mg/kg) and xylazine (1 mg/kg) until they were unresponsive to toe pinching. Then, they were orally intubated and placed on a ventilator to maintain respiration. The transverse aortic arch was accessed via an incision in the second intercostal space and surgically ligated around a 27-G needle. The needle was immediately removed after ligation. Sham group animals underwent the same procedure but without aortic ligation.

### Echocardiography

We used a Vevo 770 imaging system equipped with a 30-MHz transducer (VisualSonics, Toronto, Canada) for transthoracic echocardiography. Mice were anesthetized with an isoflurane (2%) and O_2_ (2 L/min) mixture. We measured the left ventricular (LV) posterior wall at diastole (LVPWd), LV internal dimension at diastole (LVIDd), and LVID at systole (LVIDs) in M-mode. Percentage fractional shortening (FS%) and percentage ejection fraction (EF%) were calculated as described previously [16]. We evaluated peak E, peak A, and the E/A ratio based on transmitral flow velocity variables using pulsed Doppler echocardiography. Transthoracic echocardiography was performed at baseline (week 0), week 6, and week 12 post-surgery.

### Transmission Electron Microscopy

Freshly dissected heart tissues (0.5×1×5 mm) were fixed with 2% glutaraldehyde overnight, washed three times with 0.2 M phosphate buffer, fixed with 1% osmium tetroxide, washed with 0.2 M phosphate buffer, and dehydrated in a series of ethanol concentrations. Specimens were immersed in Epon 812 resin/acetone (1∶1) for 30 min, then fresh Epon 812 resin for 30 min, and then embedded and incubated overnight at 70°C. The tissues were sectioned into 50-nm thick slices using an LKB-8800 ultramicrotome (LKB-Produkter AB, Bromma, Sweden). Cardiomyocyte mitochondria and sarcomeres were observed with an H-7000FA transmission electron microscope (Hitachi, Tokyo, Japan) at ×10000 magnification.

### Histology and Immunohistochemical Analysis

Freshly dissected heart tissues were fixed with 4% paraformaldehyde, dehydrated, and embedded in paraffin. After deparaffinization and rehydration, myocardial sections were stained with hematoxylin and eosin (HE) or Masson’s trichrome. Digital images were obtained at ×400 magnification by microscopy (Olympus, Tokyo, Japan). Single cardiomyocytes containing a nucleus were selected from HE-stained transverse sections. We counted 40 myocytes to assess the mean cross-sectional area using Image-Pro Plus 5.0 (Media Cybernetics, Houston, TX, USA). For degree of fibrosis, we analyzed 10 randomly selected frames from Masson trichrome–stained sections using Image-Pro Plus 5.0. We used primary antibodies against 8-hydroxydeoxyguanosine (8-OHdG; Abcam, Cambridge, UK) and CD31 (Santa Cruz Biotechnology, Santa Cruz, CA, USA) for immunohistochemical analysis. We counted the number of 8-OHdG–positive nuclei and CD31-positive vessels manually under ×400 magnification, and calculated the number of vessels per mm^2^ to determine the number of vessels per field. We analyzed 10 random microscopic fields from each sample.

### Malondialdehyde Assay

We assayed myocardial malonaldehyde (MDA) levels using a commercially available kit according to the manufacturer’s instructions (Jiancheng Bioengineering Institute, Nanjing, China).

### Real-time Reverse Transcription–PCR

Total RNA was extracted from heart tissue with TRIzol (Invitrogen, Carlsbad, CA, USA) and reverse-transcribed using a complementary DNA reverse transcription kit (Takara Biotechnology, Tokyo, Japan). Reactions were performed in a real-time PCR thermocycler (iQ5; Bio-Rad, Hercules, CA, USA) using SYBR green as the fluorescence dye. The mRNA expression of the target genes was normalized to the control glyceraldehyde-3-phosphate dehydrogenase (*GAPDH*) using the comparative threshold cycle (2^−ΔΔCt^) method. Table S1 lists the primer sequences used.

### Western Blotting

Total protein from heart tissues or cells was extracted using radioimmunoprecipitation lysis buffer (Beyotime, Shanghai, China). The primary antibodies used were as follows: anti-VEGF (Proteintech, Chicago, IL, USA); anti–NADPH oxidase 4 (Nox4), anti–heme oxygenase-1 (HO-1, Abcam); anti–phosphatidylinositol 3-kinase (PI3K) p85α, anti-phosphorylated (p) PI3K p85α (Tyr508) (Santa Cruz Biotechnology); anti-VEGF receptor 2 (VEGFR2), anti–p-VEGFR2 (Tyr1175), anti-Akt, anti–p-Akt (Ser473), anti–p-eNOS (Ser1177), anti-GAPDH (Cell Signaling Technology, Danvers, MA, USA); anti-eNOS (Sigma, St. Louis, MO, USA). Bands were visualized with a FluorChem E data system (Cell Biosciences, Santa Clara, CA, USA) and quantified by densitometry using Quantity One 4.52 (Bio-Rad).

### Nitrite Assay

We measured the level of nitrites (stable NO metabolites) in heart tissues spectrophotometrically using the Griess reagent system (Promega, Madison, WI, USA) as previously described [17]. Briefly, tissue samples were homogenized in buffer, and then centrifuged. The supernatant (100 µl) was incubated with 100 µl Griess reagent at room temperature for 10 min away from light. Then, the absorbance was measured at 540 nm using a microplate reader. The nitrite content was determined by comparison with the nitrite standard reference curve.

### Statistics

Data are presented as the mean ± SEM. Differences between groups were compared by one-way ANOVA. We used the Kaplan-Meier method for survival analysis; we tested between-group differences in survival using the log-rank (Mantel-Cox) test. All statistical analyses were performed using Prism 5 (GraphPad Software Inc., San Diego, CA, USA). *P*<0.05 was considered statistically significant.

## Results

### TXL Attenuated Pressure Overload–induced Cardiac Dysfunction and Mortality

**Figure 1:**
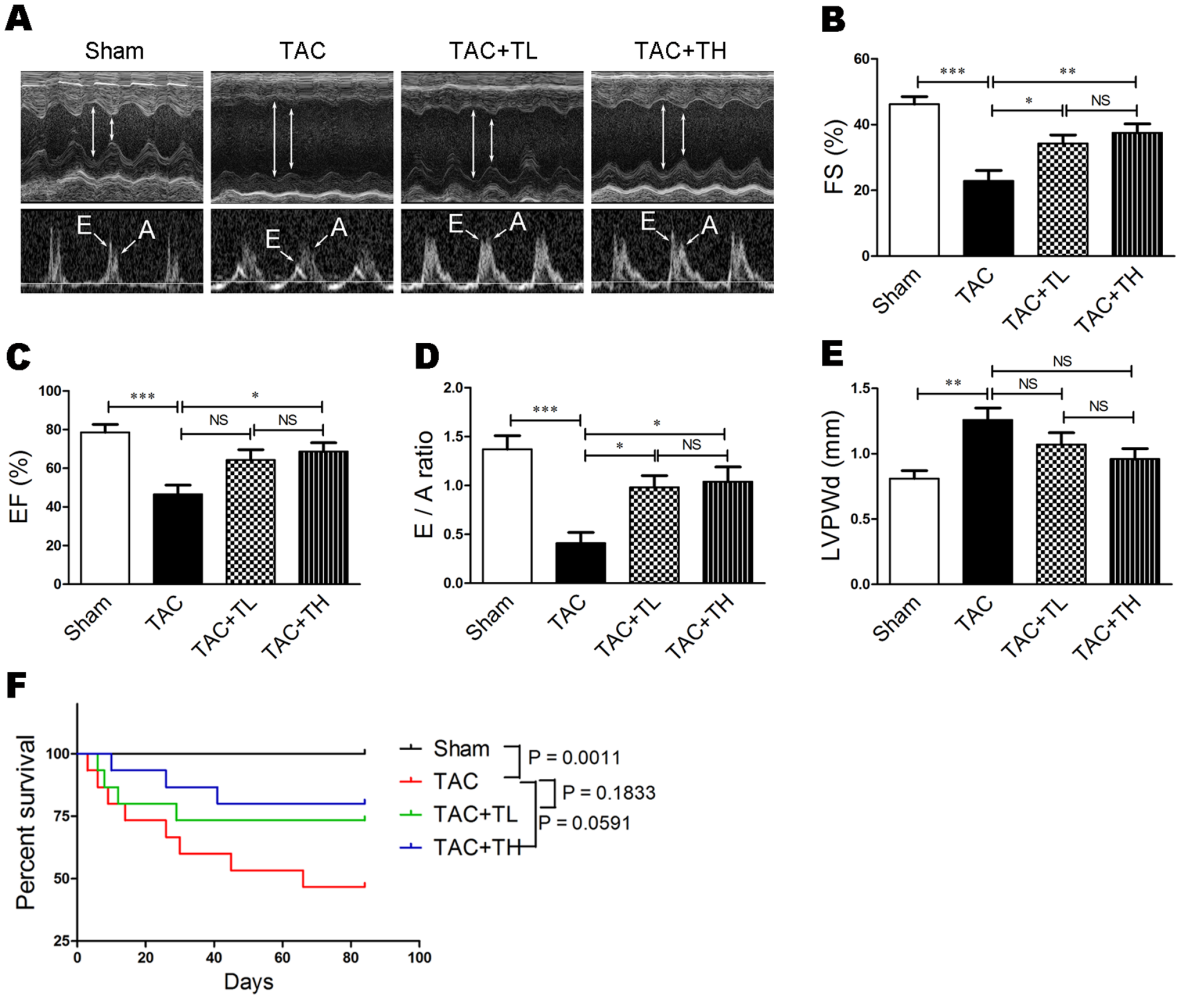
TXL improves cardiac function and reduces mortality following TAC. (**A**) Transthoracic echocardiography at the end of 12 weeks. Evaluation of (**B**) FS%, (**C**) EF%, (**D**) E/A ratio, and (**E**) LVPWd (n = 7–8 per group). (**F**) Kaplan-Meier survival curves for different groups (n = 15 per group). Data are mean ± SEM. *P<0.05, **P<0.01, ***P<0.001. Sham, sham group; TAC, mice that underwent TAC surgery without treatment; TAC+TL, mice that underwent TAC surgery treated with low-dose TXL; TAC+TH, mice that underwent TAC surgery treated with high-dose TXL; NS, not significant.

To investigate the effect of TXL on pressure overload–induced heart failure, we performed TAC surgery on mice and evaluated cardiac systolic and diastolic function by echocardiography. At six weeks post-surgery, decreased FS% was prevented in the TXL-treated groups compared with the TAC group (Table S2, *P*<0.05). At 12 weeks after surgery, the LVIDs was significantly decreased in TXL-treated mice (Table S2 and Figure 1A, *P*<0.01 or *P*<0.001) and the EF%, FS%, and E/A ratio were significantly increased (Table S2 and Figure 1A–D, *P*<0.05 or *P*<0.01) compared with the TAC group. At 12 weeks after surgery, the LVPWd increase was inhibited in TXL-treated mice compared with the TAC group, but the differences were not statistically significant (Table S2 and Figure 1E). Furthermore, at the end of the 12 weeks, mortality in the TXL-treated groups was lower than that in the TAC group (Figure 1F, *P* *= *0.1833 or *P = *0.0591). These data suggest that low- and high-dose TXL reduces TAC-induced mortality in mice.

### TXL Prevented Pressure Overload–induced Cardiac Hypertrophy

**Figure 2:**
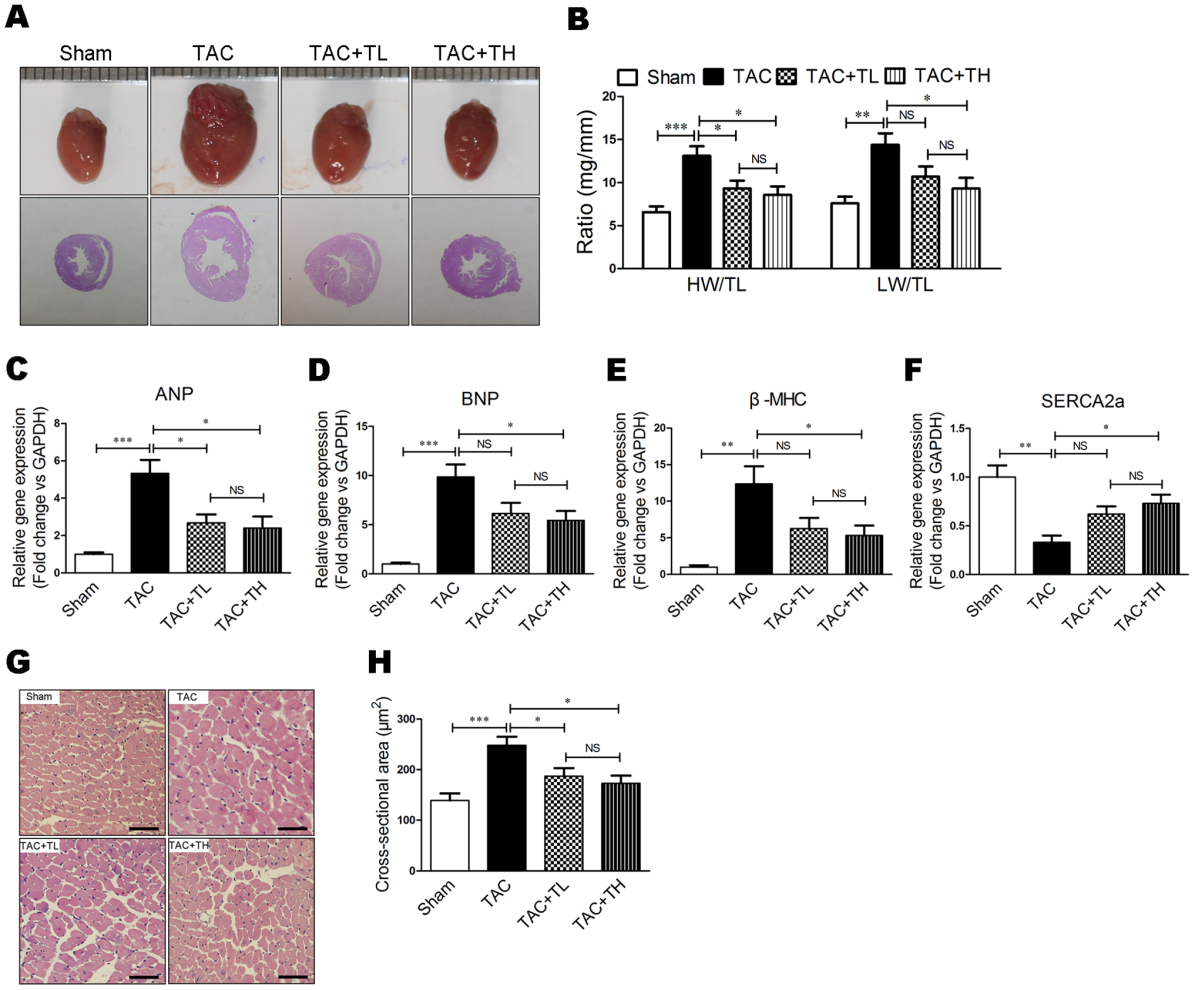
TXL prevents pressure overload–induced cardiac hypertrophy. (**A**) Representative photographs of hearts and HE staining of the hearts at 12 weeks post-surgery. (**B**) Heart weight/tibial length (HW/TL) and lung weight/tibial length (LW/TL) ratios at 12 weeks post-surgery (n = 7–8 per group). Reverse transcription–PCR (RT-PCR) of relative mRNA levels of (**C**) ANP, (**D**) BNP, (**E**) β-MHC, and (**F**) SERCA2a. (**G**) HE-stained transverse sections of left ventricles. Scale bar, 50 µm. (**H**) Quantification of cross-sectional area of cardiomyocytes from HE-stained sections (n = 5 per group). Data are mean ± SEM. *P<0.05, **P<0.01, ***P<0.001. NS, not significant.

We evaluated heart morphology and weight to assess hypertrophic remodeling following TAC. TXL inhibited cardiac dilatation and significantly reduced the heart weight/tibial length and lung weight/tibial length ratios at 12 weeks post-surgery (Figure 2A and 2B, *P*<0.05). To investigate established gene markers of cardiac hypertrophy, we analyzed myocardial mRNA expression of atrial natriuretic peptide (ANP), brain natriuretic peptide (BNP), β-myosin heavy chain (β-MHC), and sarcoplasmic reticulum Ca^2+^ adenosine triphosphatase (SERCA2a). The increase in ANP, BNP, and β-MHC was suppressed (Figure 2C–E, *P*<0.05) and the SERCA2a decrease was ameliorated in the TAC+TH group compared with the TAC group (Figure 2F, *P*<0.05). Moreover, the cardiomyocyte cross-sectional area was decreased in the TXL-treated groups compared with the TAC group (Figure 2G and 2H, *P*<0.05).

### TXL Reduced Pressure Overload–induced Cardiac Fibrosis and Ameliorated Myocardial Ultrastructure Derangement

**Figure 3:**
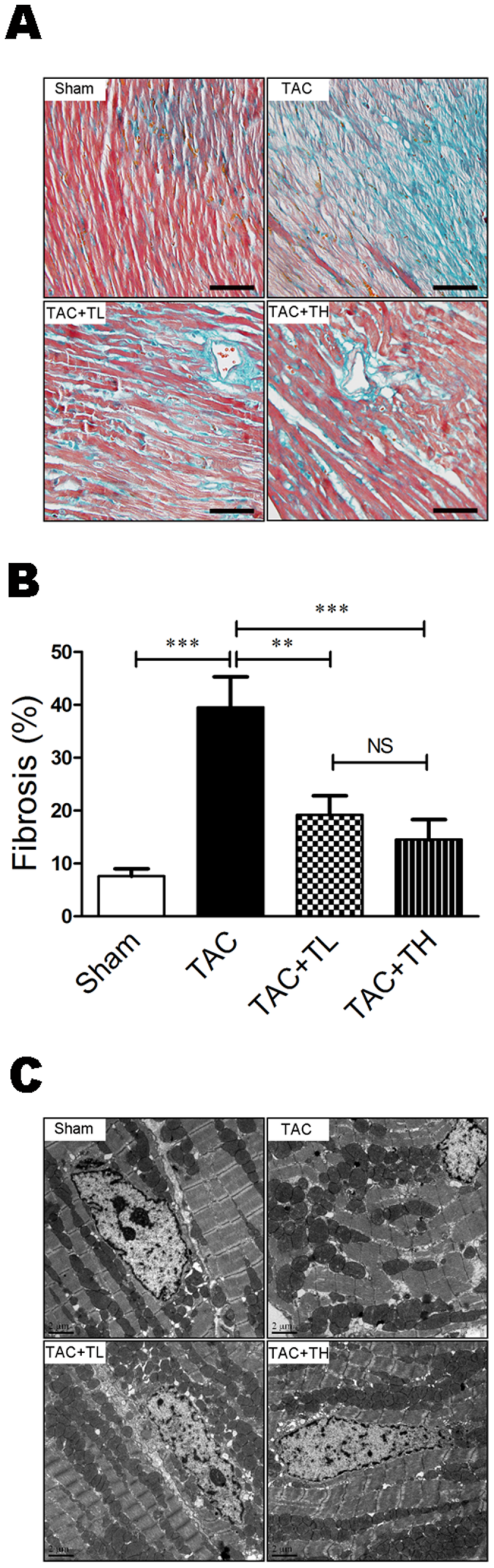
TXL reduces cardiac fibrosis and ameliorates myocardial ultrastructure derangement after TAC. (**A**) Masson trichrome–stained sections of left ventricles. Scale bar, 50 µm. (**B**) Quantification of cardiac fibrosis area from Masson trichrome–stained sections (n = 5 per group). (**C**) Transmission electron micrographs of cardiomyocytes the respective treatment groups. Scale bar, 2 µm. Data are mean ± SEM. **P<0.01, ***P<0.001. NS, not significant.

To assess TXL improvement of post-TAC cardiac fibrosis, we examined the collagen volume fraction in the LV myocardium following Masson trichrome staining. The collagen volume fraction was reduced significantly in the TAC+TL and TAC+TH groups compared with the TAC group (Figure 3A and 3B, *P*<0.01 or *P*<0.001). To assess the effect of TXL on myocardial ultrastructure, we observed mitochondrial morphology and Z-line structures using transmission electron microscopy. Failing hearts had mitochondrial morphological alteration and disorganized cristae, and disorganized Z-line structures (Figure 3C). In contrast to the TAC group, mitochondrial morphology and Z-line structures were nearly normal in the TXL-treated groups (Figure 3C).

### TXL Promoted Myocardial Capillary Density

**Figure 4:**
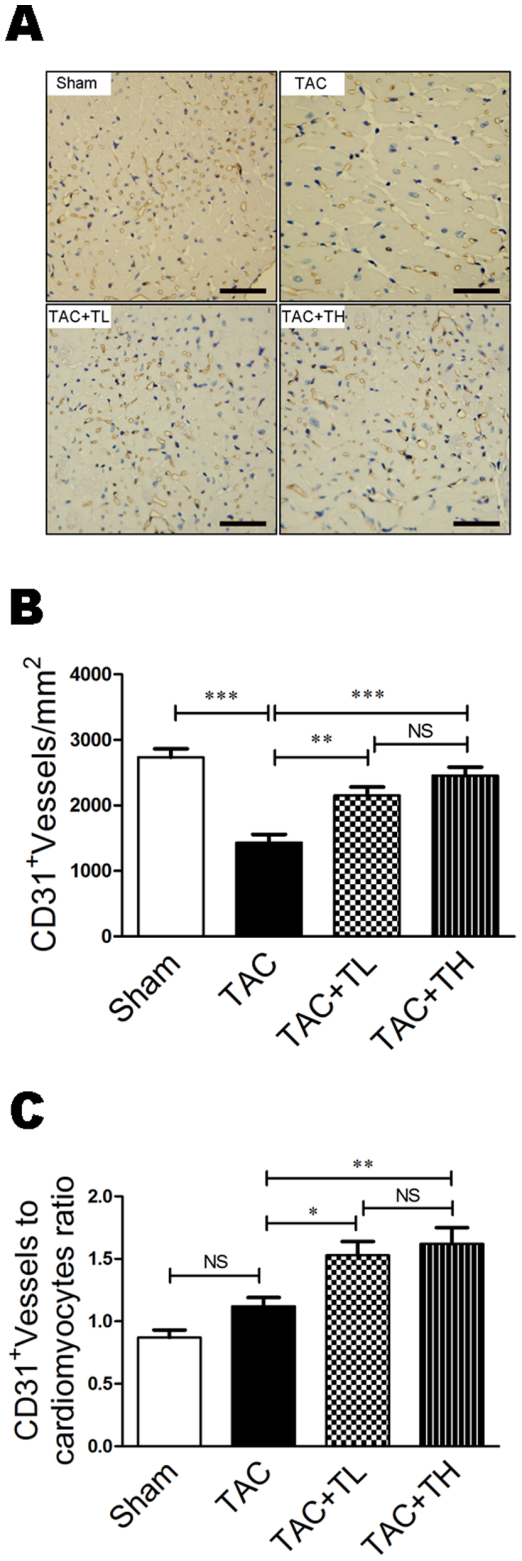
TXL promotes myocardial capillarity after TAC. (**A**) Representative immunostaining of LV myocardial capillaries (CD31+) at 12 weeks post-surgery. (**B**) Quantification of LV myocardial capillary density at 12 weeks post-surgery. (**C**) Capillary number/cardiomyocyte ratios at 12 weeks post-surgery. Data are mean ± SEM, n = 5 per group. *P<0.05, **P<0.01, ***P<0.001. NS, not significant.

To study the effects of TXL on myocardial capillarity, we investigated capillary density in the heart using CD31 immunohistochemical staining. There was a significant decrease in the number of CD31-positive vessels in failing hearts compared with hearts in the Sham group (Figure 4A and 4B, *P*<0.001). However, there was significant promotion of capillary density in the TXL-treated groups compared with the TAC group (Figure 4A and 4B, *P*<0.01 or *P*<0.001) and of the CD31-positive vessel/cardiomyocyte ratio (Figure 4C, *P*<0.05 or *P*<0.01).

### TXL Attenuated Pressure Overload–induced Cardiac Oxidative Stress Injury

**Figure 5:**
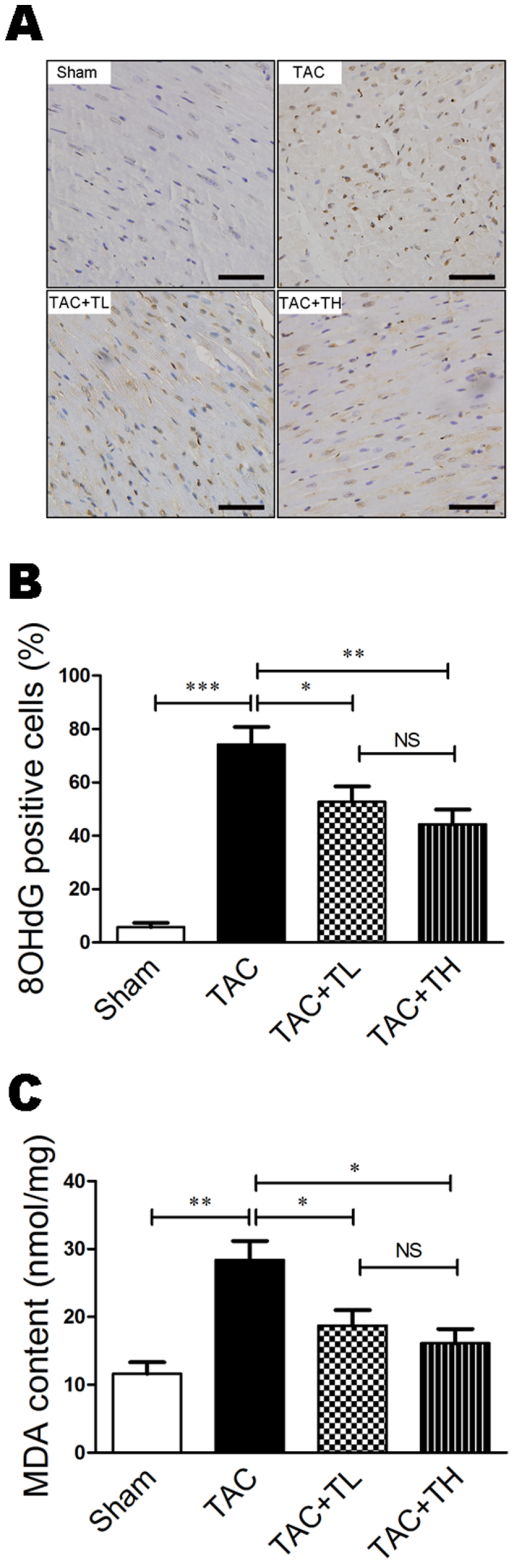
TXL attenuates 8-OHdG expression and MDA content after TAC. (**A**) 8-OHdG–immunostained sections of LV myocardium. Scale bar, 50 µm. (**B**) Quantitative analysis of the proportion of 8-OHdG–positive nuclei at 12 weeks post-surgery. (**C**) Quantification of MDA in homogenized fresh heart tissues at 12 weeks post-surgery. Data are mean ± SEM, n = 5 per group. *P<0.05, **P<0.01, ***P<0.001. NS, not significant.

Oxidative stress is considered an important cause of pressure overload–induced heart failure [18]. As MDA and 8-OHdG are cellular oxidative stress biomarkers, we detected 8-OHdG expression and MDA in the myocardium to evaluate changes in oxidative stress. MDA and 8-OHdG expression were significantly increased in failing hearts compared with the Sham group (Figure 5A–C, *P*<0.01 or *P*<0.001). In comparison to the TAC group, there was reduced accumulation of 8-OHdG–positive cells and MDA in the TXL-treated groups (Figure 5A–C, *P*<0.05 or *P*<0.01).

### TXL Augmented the VEGF/Akt/eNOS Pathway in Pressure Overload–induced Failing Hearts

**Figure 6:**
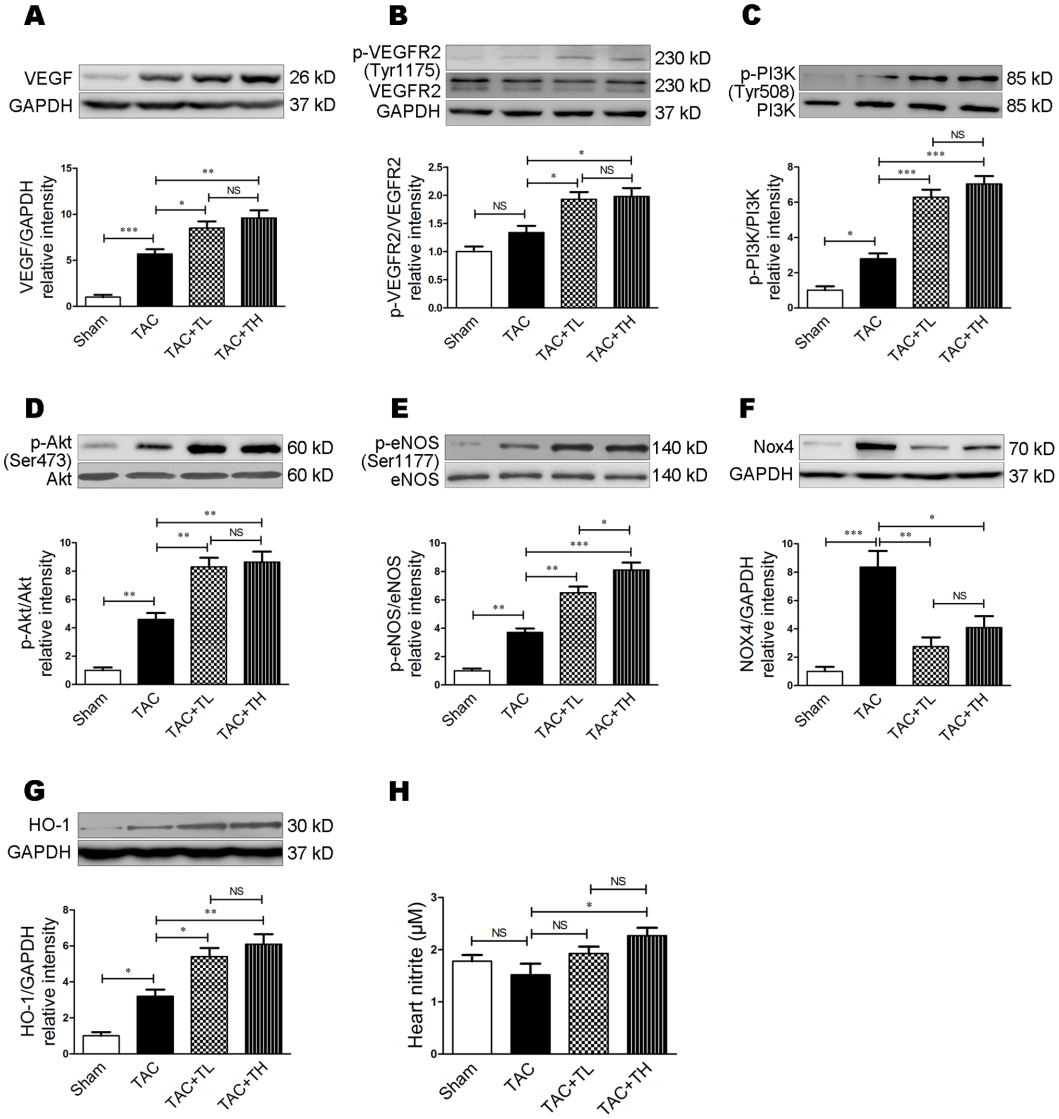
TXL activates the VEGF/Akt/eNOS pathway after TAC. Western blot analysis of (**A**) VEGF, (**B**), VEGFR2 and p-VEGFR2 (Tyr1175), (**C**), PI3K and p-PI3K (Tyr508), (**D**), Akt and p-Akt (Ser473), (**E**), eNOS and p-eNOS (Ser1177), (**F**), Nox4, and (**G**) and HO-1 expression at 12 weeks post-surgery. (**H**) Nitrite content of the respective treatment groups at 12 weeks post-surgery. Data are mean ± SEM, n = 5 per group. *P<0.05, **P<0.01, ***P<0.001. NS, not significant.

To understand the molecular mechanisms of TXL-mediated improvement of pressure overload–induced heart failure, we investigated the expression of proteins associated with angiogenesis and antioxidation. At 12 weeks post-surgery, VEGF, p-PI3K (Tyr508), p-Akt (Ser473), and p-eNOS (Ser1177) protein levels were higher in failing hearts than in Sham group hearts (Figure 6A–E, *P*<0.05, *P*<0.01, or *P*<0.001). There was increased VEGF, p-VEGFR2 (Tyr1175), p-PI3K, p-Akt, and p-eNOS protein expression in the TXL-treated groups compared to the TAC group (Figure 6A–E, *P*<0.05, *P*<0.01, or *P*<0.001). Next, we investigated the expression of Nox4 and the antioxidant HO-1. At 12 weeks post-surgery, Nox4 expression was significantly increased in the TAC group compared with the Sham group (Figure 6F, *P*<0.001). Both low- and high-dose TXL inhibited Nox4 expression significantly (Figure 6F, *P*<0.05 or *P*<0.01). Additionally, HO-1 expression was increased in the TAC group compared with the Sham group (Figure 6G, *P*<0.05). However, TXL further upregulated HO-1 expression in failing hearts compared with that in the TAC group (Figure 6G, *P*<0.05 or *P*<0.01). Furthermore, cardiac nitrite levels in the TAC+TH group were significantly higher than that in the TAC group (Figure 6H, *P*<0.05).

## Discussion

Clinically, pressure overload–induced heart failure commonly results from hypertension or aortic valvular stenosis. In the present study, we used a TAC mouse model to mimic pressure overload–induced stress. We demonstrated that TXL improved pressure overload induced–cardiac dysfunction and remodeling, promoting myocardial capillarity and attenuating oxidative stress injury. We also found that TXL upregulated cardiac nitrite content, increased VEGF, p-VEGFR2, p-PI3K, p-Akt, p-eNOS, and HO-1 expression and decreased Nox4 expression in pressure overload–induced failing hearts.

A previous study demonstrated that cardiomyocyte size and capillary density are mismatched during the development of heart failure, which results in myocardial hypoxia [19]. Crucially, cardiac angiogenesis is involved in the adaptive mechanism of cardiac hypertrophy, and inhibition of angiogenesis induces systolic dysfunction in failing hearts [20]. In the present study, cardiac capillary density was decreased in pressure overload–induced failing hearts, though the capillary/cardiomyocyte ratio was relatively increased compared with hearts in the Sham group. Promoting cardiac angiogenesis by introducing angiogenic factors restores cardiac dysfunction under chronic pressure overload [5], [20]. In this study, TXL increased capillary density and improved cardiac function in pressure overload induced–failing hearts. Thus, TXL-mediated cardiac angiogenesis may play an important role in protecting against heart failure.

Using a myocardial infarction model, we previously reported that TXL promotes angiogenesis by upregulating VEGF expression [12]. VEGF signaling plays a major role in promoting neoangiogenesis and restoring the blood supply to ischemic tissues in various pathological states, including heart failure [21]. Hypoxia-inducible factor 1α (HIF-1α) appears to be associated with increased VEGF expression by binding to a site in the promoter region of the *VEGF* gene [22]. We found that VEGF expression was upregulated in failing hearts, which is consistent with the data of Shyu *et al*. [23]. In heart failure, this upregulation might be compensatory. Nevertheless, exhaustion of VEGF release during prolonged hypoxia has been considered an important factor in heart failure [24]. VEGF has other actions, including stem cell recruitment and homing [25], decreasing apoptosis [26], and modulating autonomic response [27]. In this study, TXL significantly increased VEGF expression. Previously, we demonstrated that TXL increases the DNA-binding activity of HIF-1α [12]. Thus, we may presume that TXL promotes VEGF expression in pressure overload–induced failing hearts through HIF-1α. Moreover, there was p-VEGFR2 upregulation following TXL treatment. It is well known that VEGF exerts its angiogenic activity by binding to VEGFR2. VEGFR2 autophosphorylation and activation play a key role in vascular development and vascular permeability [28]. Our results suggest that TXL promotes VEGF-induced angiogenesis.

Akt is a serine/threonine protein kinase that regulates cardiac growth, myocardial angiogenesis, glucose metabolism, and cell death in cardiomyocytes [29]. In cardiomyocytes, short-term Akt activation improves contractile function in pressure overload–induced heart failure [30]. The Akt signaling pathway is essential in VEGF-mediated postneonatal angiogenesis [31]. In the present study, p-Akt, and the upstream kinase of Akt, p-PI3K, was increased following TXL treatment. This suggests that TXL activates the PI3K/Akt pathway in pressure overload–induced failing hearts.

In heart failure, eNOS has been recognized as an important regulator of cardiac remodeling. Following chronic pressure overload, there is greater cardiac dysfunction and LV hypertrophy in eNOS−/− mice than in wild-type mice [32]. Cardiomyocyte-specific eNOS overexpression improves LV function and reduces compensatory hypertrophy after myocardial infarction [33], [34]. TXL increases eNOS expression via the PI3K/Akt/HIF-1α signaling pathway to modulate endothelium-dependent vasodilation [35]. In the present study, TXL upregulated eNOS (Ser1177) phosphorylation in the heart, but not total eNOS, which is consistent with the findings of previous studies [12], [7]. Furthermore, high-dose TXL upregulated nitrite levels in pressure overload–induced failing hearts, which is indicative of increased NO formation following TXL treatment. These data suggest that TXL activates the VEGF/Akt/eNOS pathway in mouse heart.

Oxidative stress injury induced by the accumulation of reactive oxygen species in failing hearts plays an important role in cardiac fibrosis, hypertrophy, and mitochondrial damage [36]. eNOS-derived NO can have antioxidant signaling actions in cardiomyocytes via the transcriptional activation of HO-1 expression [37]. Sustained HO-1 upregulation in the failing heart is an important beneficial adaptation that counteracts cardiac remodeling via antioxidant, antihypertrophic, antifibrotic, and proangiogenic effects [38]. In this study, TXL upregulated HO-1 expression, which might contribute to the protective effects of TXL on cardiac fibrosis and oxidative stress injury in pressure overload–induced failing hearts. Furthermore, Nox4 expression was increased following pressure overload, and TXL decreased it significantly. Nox4 is an NADPH oxidase, a major enzyme responsible for superoxide production via the transfer of electrons across the membrane from NADPH to molecular oxygen [39]. Nox4 upregulation influences increases in mitochondrial oxidative stress directly and the consequent mitochondrial dysfunction and cell death during pressure overload–induced heart failure [40]. Hence, reduced Nox4 expression might be an additional mechanism for clarifying TXL amelioration of oxidative stress injury in mouse heart following pressure overload.

Taken together, these data suggest that TXL has a direct cardioprotective effect and affects the improvement of angiogenesis and oxidative stress injury in pressure overload–induced heart failure. These actions might contribute to improvement in the remodeling of a failing heart. The protective effects of TXL involve activation of the VEGF/Akt/eNOS signaling pathway.

## Supporting Information

Table S1 **Mouse primers used for real-time RT-PCR.** *ANP*, atrial natriuretic peptide; *BNP*, brain natriuretic peptide; *β-MHC*, β-myosin heavy chain; *SERCA2a*, sarcoplasmic reticulum Ca^2+^ adenosine triphosphatase; *GAPDH*, glyceraldehyde-3-phosphate dehydrogenase.
(DOC)

Table S2 **Echocardiographic parameters according to group.** Data are mean ± SEM, n = 7–8 per group. *P<0.05, **P<0.01, ***P<0.001 vs. Sham; †*P*<0.05, ††*P*<0.01, †††*P*<0.001 vs. TAC. LVIDd, left ventricular internal dimension at diastole; LVIDs, left ventricular internal dimension at systole; LVPWd, left ventricular posterior wall at diastole; FS, fractional shortening; EF, ejection fraction; E/A, peak E/A ratio.
(DOC)
