# Correction: Tongxinluo protects against pressure overload-induced heart failure in mice involving VEGF/Akt/eNOS pathway activation

**DOI:** 10.1371/journal.pone.0220845

**Published:** 2019-08-01

**Authors:** Bo Wang, Qing Yang, Wen-wu Bai, Yi-fan Xing, Xiao-ting Lu, Yuan-yuan Sun, Yu-xia Zhao

Following publication of this article [1], concerns were raised about the following:

- In Fig 4A, the bottom of the TAC+TL panel appears similar to the top of the TAC+TH panel.

The authors have noted that the above issue was due to an error in manuscript preparation. The authors have provided an updated Fig 4 with this correction, which incorporates replacement images for the TAC+TL and TAC+TH panels.

The raw, uncropped and unadjusted images used to generate the updated Fig 4 are included as Supporting Information (S1, S2, S3 and S4 Files). The authors indicate that the underlying data for all other figures in the manuscript are also available upon request.

The authors apologize for the errors in the published article.

**Fig 4:**
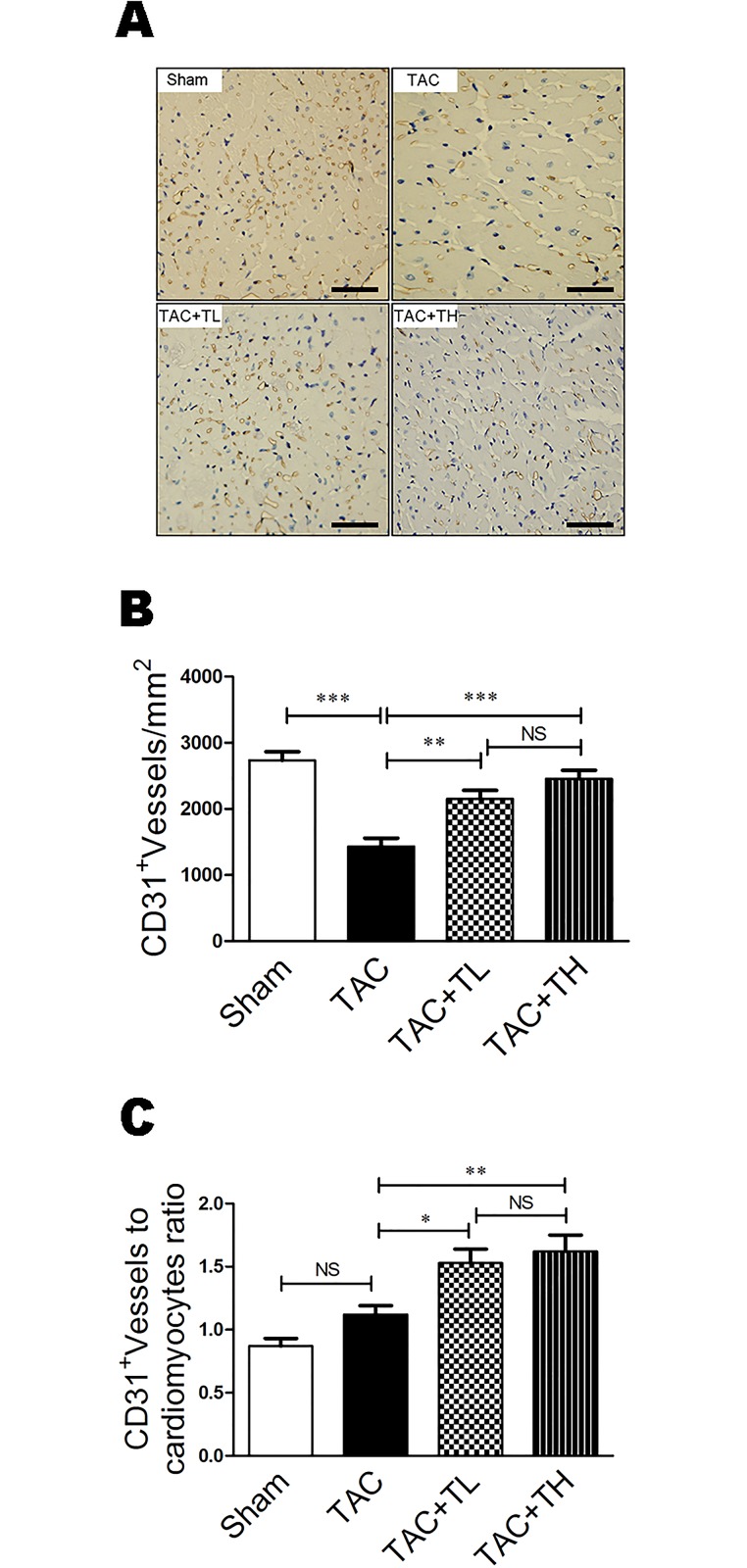
TXL promotes myocardial capillarity after TAC. (**A**) Representative immunostaining of LV myocardial capillaries (CD31+) at 12 weeks post-surgery. (**B**) Quantification of LV myocardial capillary density at 12 weeks post-surgery. (**C**) Capillary number/cardiomyocyte ratios at 12 weeks post-surgery. Data are mean ± SEM, n = 5 per group. *P<0.05, **P<0.01, ***P<0.001. NS, not significant.

## Supporting information

S1 File Sham. Raw, uncropped and unadjusted image used to generated the Sham panel of Fig 4A.
(TIF)

S2 File TAC. Raw, uncropped and unadjusted image used to generated the TAC panel of Fig 4A.
(TIF)

S3 File TAC+TL. Raw, uncropped and unadjusted image used to generated the TAC+TL panel of Fig 4A.
(TIF)

S4 File TAC+TH. Raw, uncropped and unadjusted image used to generated the TAC+TH panel of Fig 4A.
(TIF)
