# Supplementary material for: Tongxinluo Protects against Pressure Overload–Induced Heart Failure in Mice Involving VEGF/Akt/eNOS Pathway Activation
Source: PLoS One. 2014 Jun 2;9(6):e98047. doi: 10.1371/journal.pone.0098047 (PMC4041651; doi:10.1371/journal.pone.0098047)
Supplement: Table S1 — Mouse primers used for real-time RT-PCR. ANP, atrial natriuretic peptide; BNP, brain natriuretic peptide; β-MHC, β-myosin heavy chain; SERCA2a, sarcoplasmic reticulum Ca2+ adenosine triphosphatase; GAPDH, glyceraldehyde-3-phosphate dehydrogenase. (DOC) [file pone.0098047.s001.doc]

**Table S**1. Mouse primers used for real-time RT-PCR.

| Gene |  | Sequence |
| --- | --- | --- |
| *ANP* | Forward | 5′-GGACTAGGCTGCAACAGCTTC-3′ |
|  | Reverse | 5′-GTGACACACCACAAGGGCTTA-3′ |
| *BNP* | Forward | 5′-CGTCAGTCGTTTGGGCTGTAA-3′ |
|  | Reverse | 5′-CACTTCAAAGGTGGTCCCAGAG-3′ |
| *β-MHC* | Forward | 5′-ATGTGCCGGACCTTGGAA-3′ |
|  | Reverse | 5′-CCTCGGGTTAGCTGAGAGATCA-3′ |
| *SERCA2a* | Forward | 5′-CTGTGGAGACCCTTGGTTGT-3′ |
|  | Reverse | 5′-CAGAGCACAGATGGTGGCTA-3′ |
| *GAPDH* | Forward | 5′-TGTGTCCGTCGTGGATCTGA-3′ |
|  | Reverse | 5′-TTGCTGTTGAAGTCGCAGGAG-3′ |

*ANP*, atrial natriuretic peptide; *BNP*, brain natriuretic peptide; *β-MHC*, β-myosin heavy chain; *SERCA2a*, sarcoplasmic reticulum Ca^2+^ adenosine triphosphatase; *GAPDH*, glyceraldehyde-3-phosphate dehydrogenase.
