# Supplementary material for: Tongxinluo Protects against Pressure Overload–Induced Heart Failure in Mice Involving VEGF/Akt/eNOS Pathway Activation
Source: PLoS One. 2014 Jun 2;9(6):e98047. doi: 10.1371/journal.pone.0098047 (PMC4041651; doi:10.1371/journal.pone.0098047)
Supplement: Table S2 — Echocardiographic parameters according to group. Data are mean ± SEM, n = 7–8 per group. *P<0.05, **P<0.01, ***P<0.001 vs. Sham; †P<0.05, ††P<0.01, †††P<0.001 vs. TAC. LVIDd, left ventricular internal dimension at diastole; LVIDs, left ventricular internal dimension at systole; LVPWd, left ventricular posterior wall at diastole; FS, fractional shortening; EF, ejection fraction; E/A, peak E/A ratio. (DOC) [file pone.0098047.s002.doc]

**Table S2. Echocardiographic parameters according to group.**

|  | Sham | TAC | TAC+TL | TAC+TH |
| --- | --- | --- | --- | --- |
| Week 0 |  |  |  |  |
| LVIDd (mm) | 2.98 ± 0.13 | 2.88 ± 0.12 | 3.18 ± 0.13 | 3.15 ± 0.12 |
| LVIDs (mm) | 1.58 ± 0.11 | 1.46 ± 0.09 | 1.66 ± 0.11 | 1.66 ± 0.10 |
| LVPWd (mm) | 0.72 ± 0.05 | 0.77 ± 0.06 | 0.72 ± 0.06 | 0.73 ± 0.05 |
| FS (%) | 47.2 ± 2.5 | 49.6 ± 2.3 | 47.8 ± 2.3 | 47.4 ± 2.2 |
| EF (%) | 79.9 ± 3.3 | 82.1 ± 4.2 | 80.4 ± 3.9 | 80.0 ± 3.4 |
| E/A | 1.53 ± 0.12 | 1.61 ± 0.14 | 1.49 ± 0.13 | 1.57 ± 0.13 |
| Week 6 |  |  |  |  |
| LVIDd (mm) | 3.37 ± 0.14 | 3.41 ± 0.16 | 3.73 ± 0.14 | 3.54 ± 0.13 |
| LVIDs (mm) | 1.77 ± 0.11 | 2.31 ± 0.13* | 2.18 ± 0.12 | 2.03 ± 0.12 |
| LVPWd (mm) | 0.76 ± 0.06 | 1.12 ± 0.08** | 0.96 ± 0.07 | 0.92 ± 0.06 |
| FS (%) | 47.4 ± 2.3 | 32.2 ± 2.5*** | 41.6 ± 2.1† | 42.7 ± 2.2† |
| EF (%) | 79.7 ± 3.7 | 61.4 ± 5.3 | 73.3 ± 5.2 | 74.4 ± 4.8 |
| E/A | 1.41 ± 0.13 | 0.79 ± 0.12** | 1.06 ± 0.11 | 1.12 ± 0.14 |
| Week 12 |  |  |  |  |
| LVIDd (mm) | 3.14 ± 0.13 | 3.94 ± 0.14** | 3.56 ± 0.16 | 3.48 ± 0.15 |
| LVIDs (mm) | 1.67 ± 0.12 | 3.04 ± 0.13*** | 2.34 ± 0.15†† | 2.17 ± 0.13††† |
| LVPWd (mm) | 0.81 ± 0.06 | 1.26 ± 0.09** | 1.07 ± 0.09 | 0.96 ± 0.08 |
| FS (%) | 46.22 ± 2.3 | 22.86 ± 3.2*** | 34.27 ± 2.6† | 37.54 ± 2.7†† |
| EF (%) | 78.6 ± 4.1 | 46.5 ± 4.8*** | 64.3 ± 5.3 | 68.7 ± 4.5† |
| E/A | 1.37 ± 0.14 | 0.41 ± 0.11*** | 0.98 ± 0.12† | 1.04 ± 0.15† |

Data are mean ± SEM, n = 7-8 per group. **P* < 0.05, ***P* < 0.01, ****P* < 0.001 vs. Sham; †*P* < 0.05, ††*P* < 0.01, †††*P* < 0.001 vs. TAC. LVIDd, left ventricular internal dimension at diastole; LVIDs, left ventricular internal dimension at systole; LVPWd, left ventricular posterior wall at diastole; FS, fractional shortening; EF, ejection fraction; E/A, peak E/A ratio.
