# Supplementary figures and images for: Correction: Tongxinluo protects against pressure overload-induced heart failure in mice involving VEGF/Akt/eNOS pathway activation
Source: PLoS One. 2019 Aug 1;14(8):e0220845. doi: 10.1371/journal.pone.0220845 (PMC6675059; doi:10.1371/journal.pone.0220845)

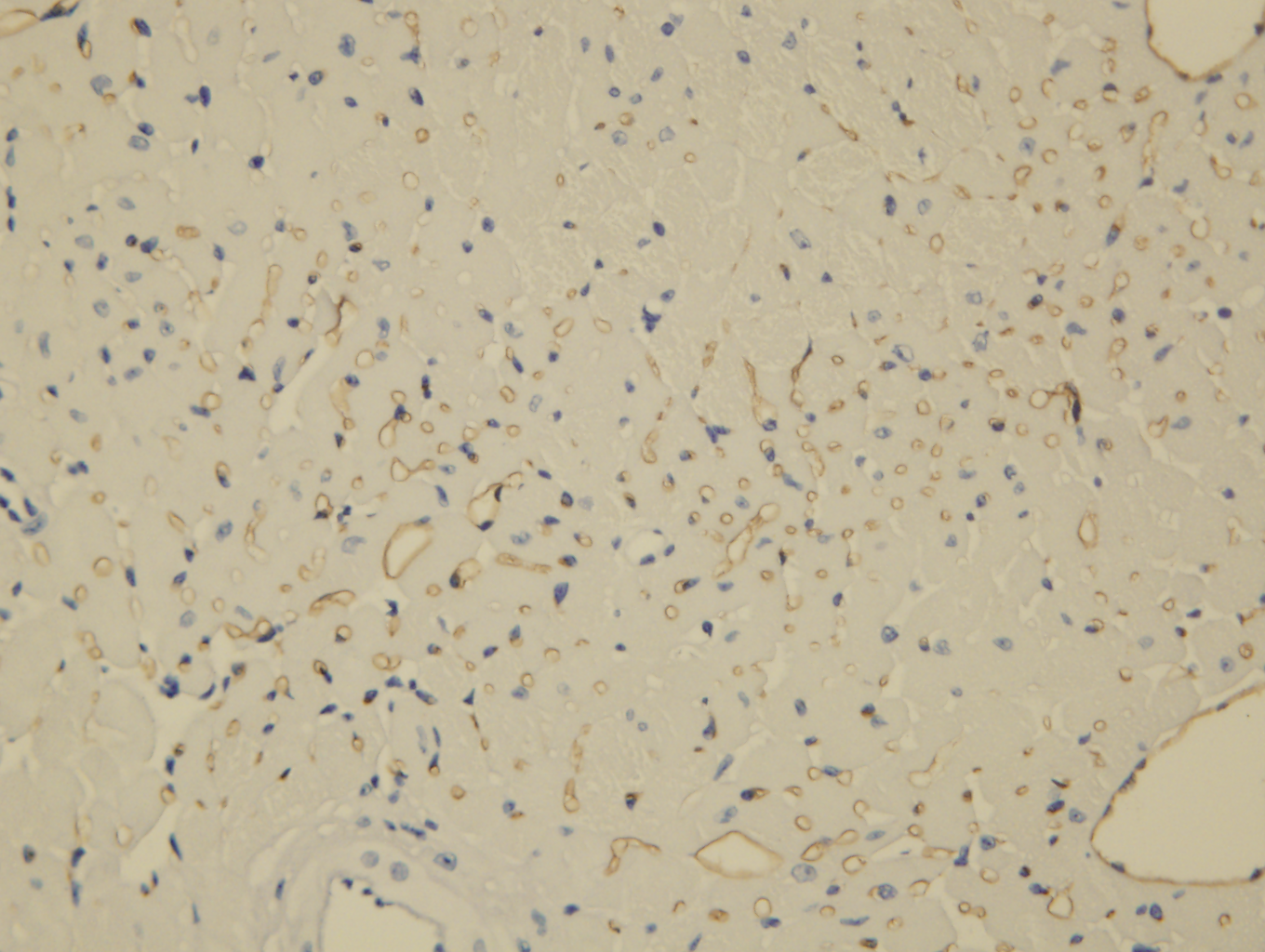

Supplement: S1 File — Raw, uncropped and unadjusted image used to generated the Sham panel of Fig 4A. (TIF) [file pone.0220845.s001.tif]

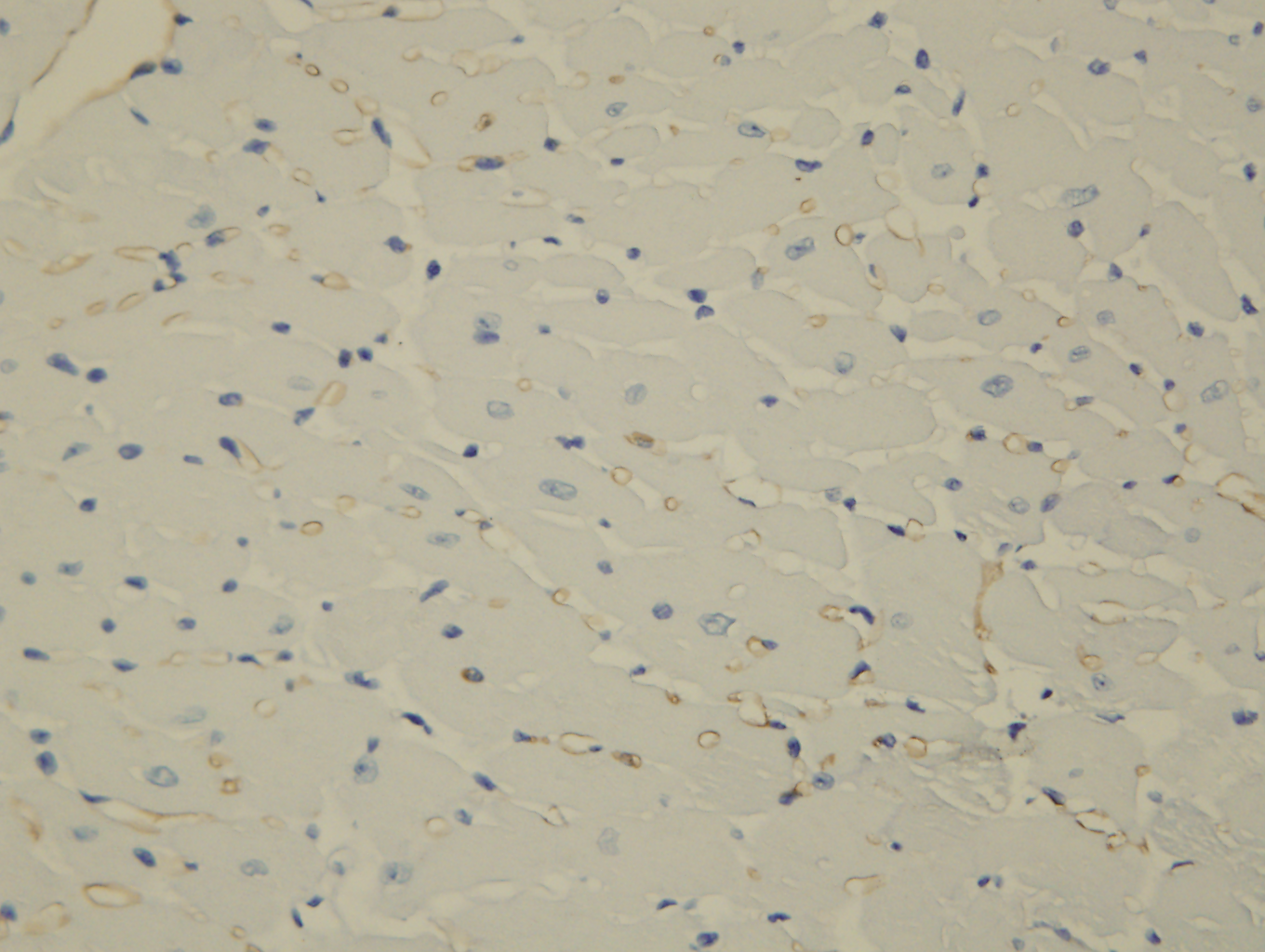

Supplement: S2 File — Raw, uncropped and unadjusted image used to generated the TAC panel of Fig 4A. (TIF) [file pone.0220845.s002.tif]

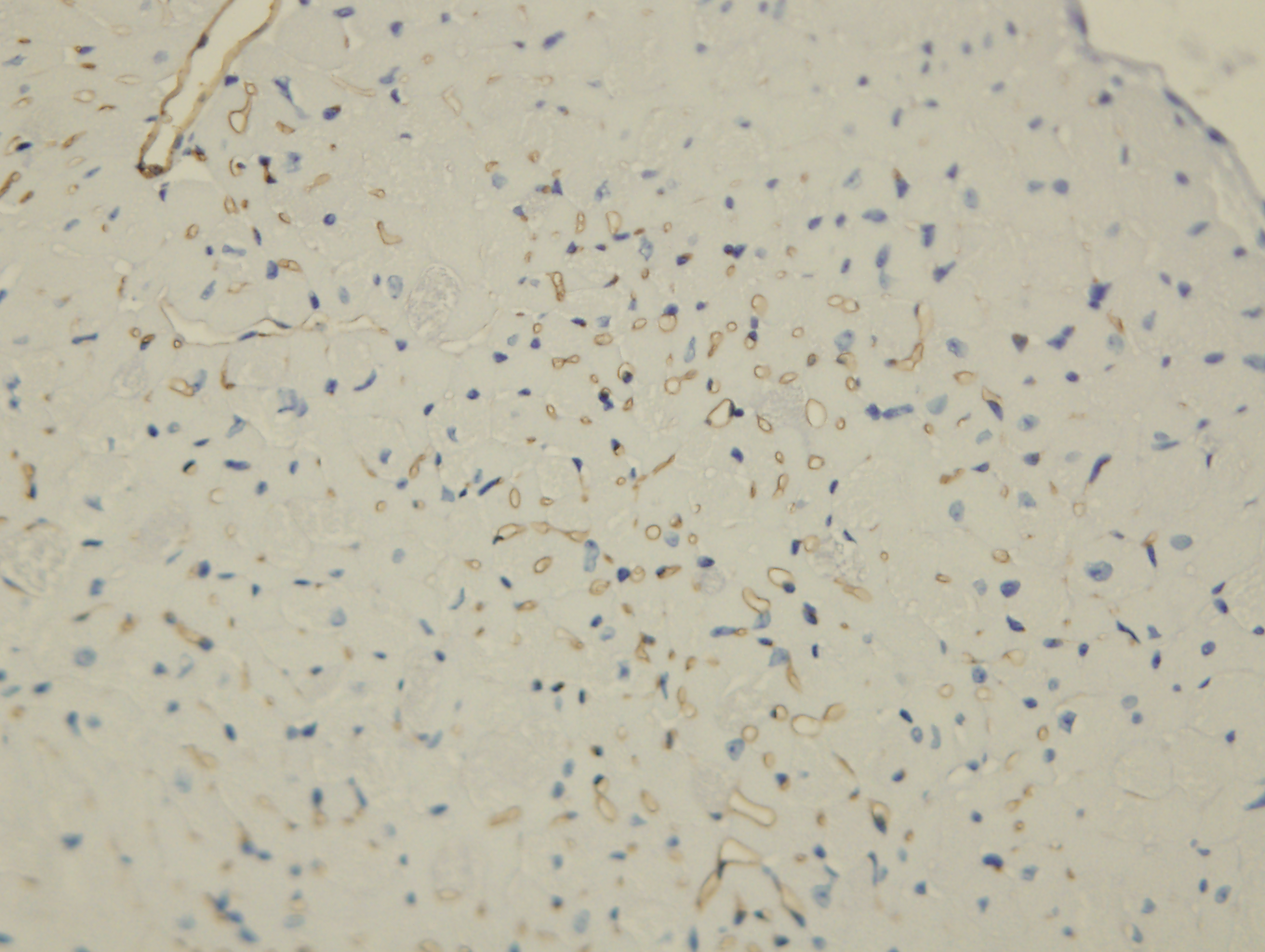

Supplement: S3 File — Raw, uncropped and unadjusted image used to generated the TAC+TL panel of Fig 4A. (TIF) [file pone.0220845.s003.tif]

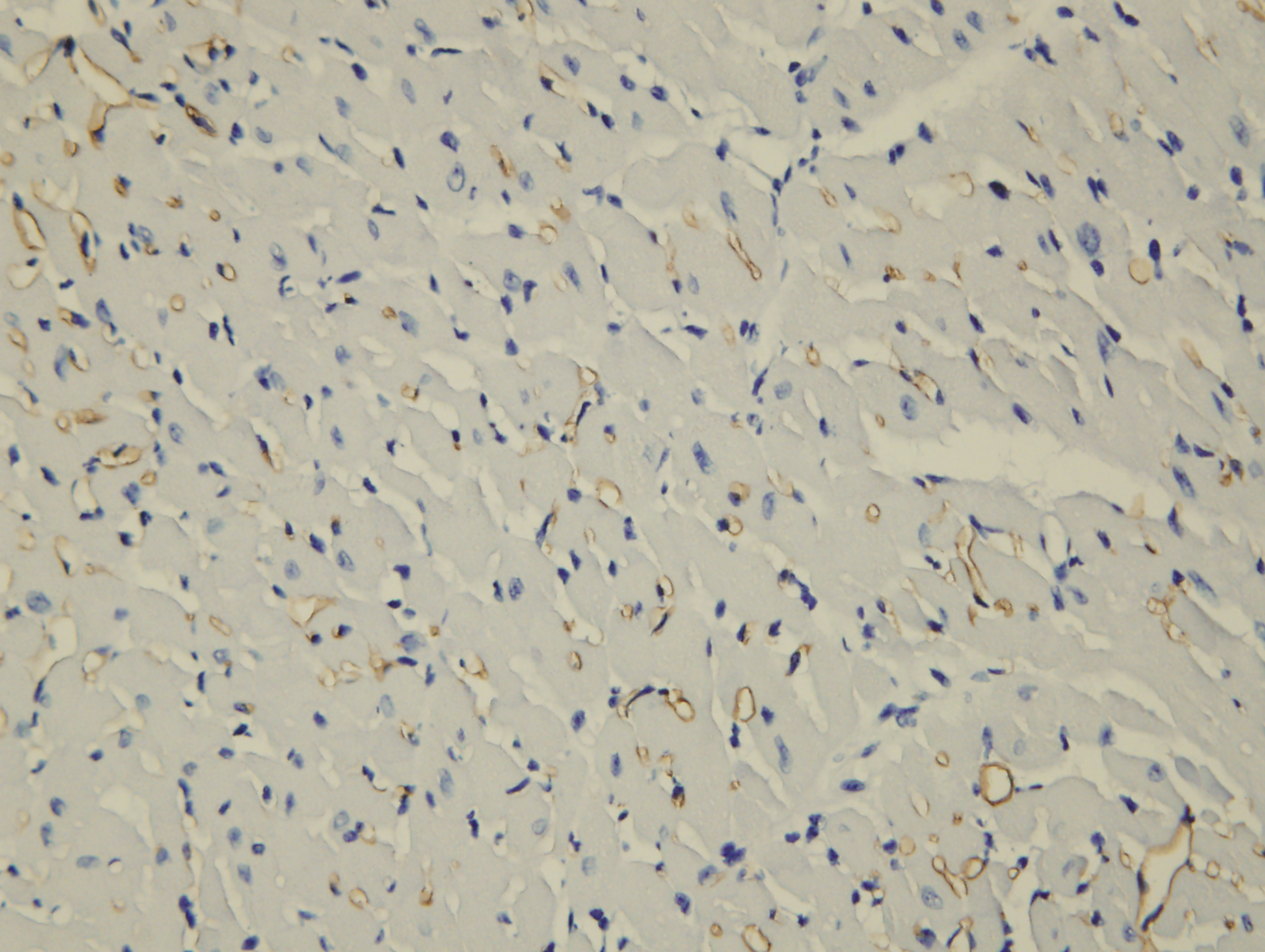

Supplement: S4 File — Raw, uncropped and unadjusted image used to generated the TAC+TH panel of Fig 4A. (TIF) [file pone.0220845.s004.tif]
